# Proteomics of Eosinophil Activation

**DOI:** 10.3389/fmed.2017.00159

**Published:** 2017-09-29

**Authors:** Deane F. Mosher, Emily M. Wilkerson, Keren B. Turton, Alexander S. Hebert, Joshua J. Coon

**Affiliations:** ^1^Department of Biomolecular Chemistry, University of Wisconsin, Madison, WI, United States; ^2^Department of Medicine, University of Wisconsin, Madison, WI, United States; ^3^Department of Chemistry, University of Wisconsin, Madison, WI, United States

**Keywords:** eosinophils, mass spectrometry-based proteomics, phosphorylation sites, interleukin-5, STAT3, integrins, immunoproteasome

## Abstract

We recently identified and quantified >7,000 proteins in non-activated human peripheral blood eosinophils using liquid chromatography coupled to tandem mass spectrometry (LC–MS/MS) and described phosphoproteomic changes that accompany acute activation of eosinophils by interleukin-5 (IL5) (1). These data comprise a treasure trove of information about eosinophils. We illustrate the power of label-free LC–MS/MS quantification by considering four examples: complexity of eosinophil STATs, contribution of immunoproteasome subunits to eosinophil proteasomes, complement of integrin subunits, and contribution of platelet proteins originating from platelet–eosinophil complexes to the overall proteome. We describe how isobaric labeling enables robust sample-to-sample comparisons and relate the 220 phosphosites that changed significantly upon treatment with IL5 to previous studies of eosinophil activation. Finally, we review previous attempts to leverage the power of mass spectrometry to discern differences between eosinophils of healthy subjects and those with eosinophil-associated conditions and point out features of label-free quantification and isobaric labeling that are important in planning future mass spectrometric studies.

## Introduction

Eosinophils derive from precursors set aside early in hematopoietic differentiation (2) and are easily identified in a Giemsa-stained blood smear by their abundant plump red granules and bilobed nucleus. Eosinophils have nuanced roles in normal physiology and responses to injury or pathogenic agents (3, 4), contributing to tissue homeostasis in the gut and adipose tissue and featuring prominently in inflammation associated with allergic diseases, malignancies, viral and helminthic infections, and orderly tissue repair (4–8). Eosinophils have the potential to participate in the pathogenesis of disease by diverse mechanisms, including release of a unique set of granule components, secretion of cytokines, and elaboration of mediators (3, 4). The need for better understanding of eosinophils in the context of eosinophil-associated diseases was highlighted in the report of a taskforce assembled by the National Institutes of Health (9).

Eosinophils were not among the >200 tissues, cell lines, and purified cell populations analyzed to assemble draft human proteomes published in 2014 (10, 11). We recently reported two high-resolution mass spectrometric investigations of human peripheral blood eosinophils: (1) identification and quantification of the proteins of non-activated eosinophils and (2) description of phosphoproteomic changes that accompany acute activation by interleukin-5 (IL5) (1). These data represent important information about eosinophils. An explicit goal of this review is to facilitate access to and increase the usefulness of the data presented in supplementary spreadsheets of our paper.

## Quantitative Analysis of the Eosinophil Proteome

The workhorse of modern global proteomics is reversed-phase liquid chromatographic (LC) separation of proteolytically generated peptides coupled to online nano-electrospray ionization of the effluent and identification of peptide mass/charge (*m*/*z*) and sequence by tandem mass spectrometry (MS/MS) (12). Peptide cations detected in the MS^1^ scan are subjected to dissociation and subsequent MS^2^ scan, yielding an ion series that can indicate amino acid sequence and the presence of posttranslational modification. The resulting spectra are compared with peptides generated *in silico* to determine which of the ~2 × 10^4^ encoded human proteins and many more proteoforms (13) arising from differential mRNA splicing are present in the sample based on peptide spectral matches. To increase the probability of identification of any given peptide, peptides can be fractionated by a preliminary LC separation after which each fraction is analyzed in a separate liquid chromatography coupled to tandem mass spectrometry (LC–MS/MS) run. This paradigm routinely identifies thousands of proteins and proteoforms from biological samples (10–12). Estimates of relative abundance of identified proteins, called label-free quantification (LFQ), can be made by intensity-based absolute quantification (iBAQ), which sums signal intensities of all identified peptides for a given protein and divides by the number of theoretically observable peptides based on the *in silico* digest (14). The technology has been improving continuously to increase proteome coverage, speed of analysis, and quality of data with the goal of increasing applicability to biological experimentation and clinical samples. For instance, our group is able to quantify nearly 90% of the estimated 4,500 proteins in the yeast proteome in ~1 h of analysis (12, 15).

We assembled a map of the proteome of 75 × 10^6^ non-activated peripheral blood eosinophils pooled from three different human volunteer donors with allergic rhinitis or asthma (1). Heparinized blood, 200 mL, was obtained from each, granulocytes were isolated by centrifugation in a Percoll gradient, and eosinophils were isolated from the granulocyte fraction by negative selection with magnetic beads bearing antibodies to CD3, CD14, CD16, and glycophorin-A (16, 17). Cells were lysed *via* probe sonication in a urea buffer, and proteins were digested with trypsin. Phosphopeptides were enriched by immobilized metal affinity chromatography (IMAC). Non-enriched and enriched samples were separated by high pH reversed-phase chromatography into 30 and 20 fractions, respectively, and fractions were analyzed by LC–MS/MS on an Orbitrap Fusion. The UniProt human proteins-plus-proteoforms database as of April 4, 2014, was queried using MaxQuant with the Andromeda search engine that included the iBAQ algorithm (14, 18, 19), yielding iBAQ intensities that can be translated into absolute molar abundances by assuming direct proportionality.

We identified 7,086 proteins based on 100,892 different tryptic peptides (1). Estimates of cellular abundance correlated well with the intensities of the protein spots seen in the two-dimensional gels of an earlier published proteomic study (20), with actin being the most abundant protein in both. The 15 most abundant proteins accounted for 25% of protein molecules. These include the granule proteins RNASE2 (eosinophil-derived neurotoxin), RNASE3 (eosinophil cationic protein), C-terminal remnant of PRG2 (major basic protein 1), C-terminal remnant of PRG3 (major basic protein 2), and CLC (Charcot-Leyden crystal protein, galectin 10); proteins associated with actin cytoskeleton (ACTB, PFN1, and CFL1); and histones. The abundances (molecules per eosinophil) of RNASE2 (1.8 × 10^8^), RNASE3 (2.5 × 10^8^), PRG2 (6.4 × 10^8^), and eosinophil peroxidase (1.7 × 10^8^) previously had been quantified by radioimmunoassay (21), thus allowing calculations of the absolute abundances of other proteins. The iBAQ intensities in Sheet 1 of the paper’s supporting XLSL file entitled “Summary of proteins identified in global analysis… ordered from most to least abundant” ranged from 1.3 × 10^11^ for ACTB (cytoplasmic actin) to 3.1 × 10^3^ for KIAA1211 (1). The ratios of the iBAQ intensities to cellular abundances of the four granule proteins average 360, and division by this number can be used to convert iBAQ score to molecules per cell. We also localized 4,802 sites of phosphorylation as described in the paper’s supporting XLSL file entitled “Summary of phosphosites identified in global analysis…” (1).

Selected entries from the “global analysis” file have been pasted into Sheet 1 of the XLSX in the supplement of this review. A single entry may describe a single protein or a group that may consist of proteins of the same or nearly identical sequence encoded by separate genes, as for several of the histones; different proteoforms encoded by a single gene; or a single proteoform. In addition, frequently peptides will be matched to several entries in a protein database rather than to a single group (22). Each entry, therefore, contains information about rank in abundance; UniProt ID(s) of all proteins and majority proteins in the group; protein names(s); gene name(s); number of proteins in the group; number of peptides matching the group; number of peptides defined as “razor,” i.e., specific for the protein group, and “unique,” i.e., specific for a given proteoform within the group; % sequence covered by the identified peptides; molecular weight and sequence length of the longest proteoform within the group; posterior error probability of misidentification of the protein group; sum of peptide ion intensities; and iBAQ intensity score.

To drill down and exploit this information, one needs to consult UniProt^1^ and, because UniProt is uneven in its annotation of possible proteoforms, one may need also to consult the literature and transcriptomic and genomic databases and perhaps to perform directed experiments. We illustrate such issues with the entries on eosinophil STATs in Sheet 1. STAT2, STAT5A, STAT5B, and STAT6 are encoded by separate genes, and each has a single entry. The entries for STAT2, STAT5A, and STAT6 describe groups of two or three proteoforms differentially spliced at or near the N-terminus that cannot be distinguished by the available proteomic data. STAT5A and STAT5B share sequence similarity such that of the identified peptides, 23 are assigned to both, 13 are unique to STAT5B, and 11 are unique to STAT5A. STAT1 and STAT3 each have two entries that describe differentially spliced proteoforms originating from single genes. For STAT1, the dominant proteoform was canonical 750-residue STAT1α, and the minor proteoform was 712-residue STAT1β with a truncated C-terminus due to a frameshift introduced by splicing. The analysis identified four peptides unique for STAT1α and one unique for STAT1β. The dominant proteoform of STAT3 was 770 residues in length, and the minor proteoform was 769 residues; these each have a single unique peptide in which Ser701 is present (S) or absent (ΔS). STAT3, such as STAT1, is subjected to splicing that generates α and β proteoforms, as was first observed at the transcript level in eosinophils (23). However, our analysis did not identify a peptide spectral match unique for STAT3β. The splicing events responsible for inclusion (S) or exclusion (ΔS) of the codon for Ser701 and the α or β variants are close to one another, such that we were able to use quantitative PCR to demonstrate the presence and proportions of the four possible STAT3 transcripts, S-α, ΔS-α, S-β, and ΔS-β, in eosinophils (24). In accord with the iBAQ data, ΔS-encoding transcripts were in the minority. We note that even if a tryptic peptide defining the β variants had been detected, we would not have known whether the peptide was derived from the S or ΔS variant or a mixture. Examination of the amino acid sequences of the four splice variants, however, indicates that such information likely could be obtained by substituting AspN protease for trypsin. AspN should generate four different peptides that span the sequences determined by the two splicing events.

The iBAQ intensities in Sheet 1 inform thinking about the complexity inherent in signaling by different eosinophil STATs. The intensities and hence abundances of STAT1, STAT3, and STAT5B are similar, with approximately 600,000 copies of each protein per eosinophil based on comparisons to the iBAQ intensities of the four granular proteins. STAT6 and STAT2 were present at approximately 70 and 12% the abundances of the three major STATs but at greater abundance than STAT5A. The complete “global analysis” file (1) allows comparisons of the abundances of numerous other classes of eosinophil proteins that have similar and perhaps overlapping functions, such as the tyrosine kinases that activate STATs.

The other entries in Sheet 1 concern proteasome subunit beta-type (PSMB) subunits of the 20S proteasome and illustrate the power of quantitative proteomics in dealing with complexes with known structure and stoichiometry. Such complexes account for a considerable fraction of the proteome (25) and are described on the CORUM website.^2^ The PSMB5, PSMB6, and PSMB7 subunits of the constitutive 20S proteasome are replaced by the PSMB8, PSMB9, and PSMB10 subunits of immunoproteasomes in T-cells and monocytes (11). The switch involves the three catalytic proteasome subunits and results in preferential generation of peptides with a hydrophobic C-terminus that can be processed to fit in the groove of MHC class I molecules (26). The ratios of iBAQ intensities of PSMB8/PSMB5, PSMB9/PSMB6, and PSMB10/PSMB7 for eosinophils are 18, 3.7, and 2.1, respectively, comparable to the values of 31, 2.9, and 2.1 reported for monocytes in ProteomicsDB^3^ [an easily navigated repository of human proteomics data (10)]. The comparable enrichment in immunoproteasome subunits in monocytes and eosinophils bears on the issue of whether eosinophils are important antigen-presenting cells (27).

**Figure 1:**
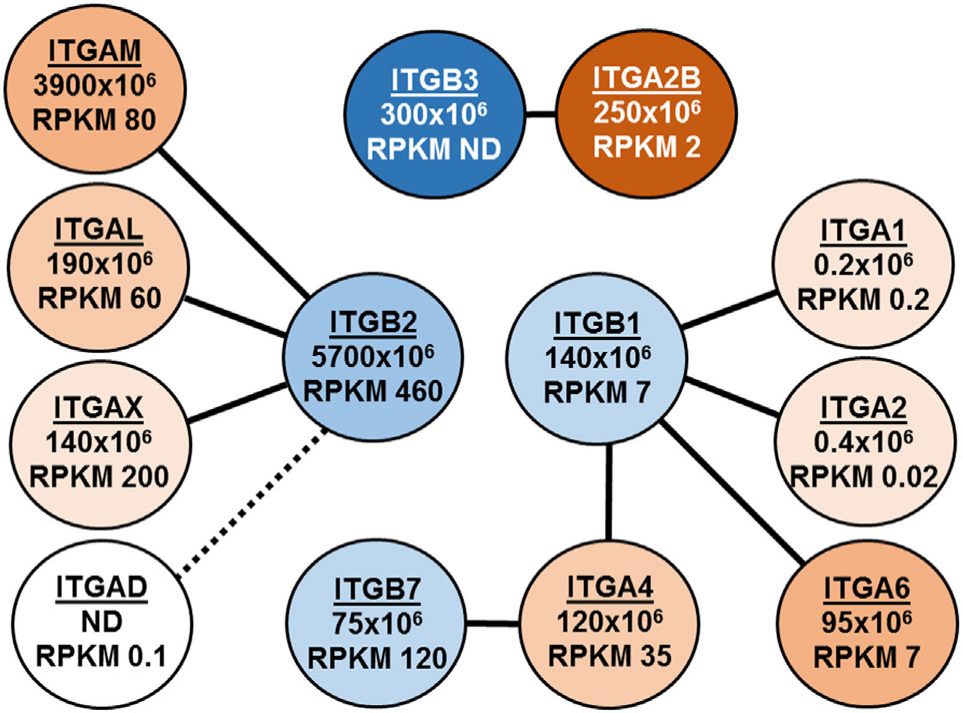
Depiction of integrin expression in eosinophils as assessed by intensity-based absolute quantification (iBAQ) intensities and RNA-Seq. Lines connect the nine αβ dimers that are possible between these subunits, and iBAQ intensities and mRNA abundance as RPKM (reads per kilobase per million mapped reads) are given.

Figure 1 illustrates a second example of insights to be gained from quantitative proteomics. Shown are our data and recently published transcriptomic RNA-Seq data (28) for the eight α-integrin (ITGA) and four β-integrin (ITGB) subunits detected in eosinophils. Lines connect the nine αβ dimers (29) that are possible between these subunits, and iBAQ intensities and mRNA abundance as RPKM (reads per kilobase per million mapped reads) are given (Figure 1). Several features are noteworthy. First, the iBAQ intensities are compatible with the proposed pairing of dimers. Second, the iBAQ intensities in general correlate with mRNA abundance. Third, protein and mRNA are missing for ITGAD, which is inconsistent with the prevailing view that there is a pool of αDβ2 that can be mobilized acutely to the eosinophil surface (29, 30). Fourth, the ITGA2B and ITGB3 subunits of αIIbβ3, the major integrin of platelets, are abundant as proteins but not as mRNA.

### Issue of Contaminating Platelets

Platelets, which adhere to a fraction of circulating eosinophils (31, 32), carry an idiosyncratic mix of RNAs (33). To investigate whether the higher-than-expected abundance of ITGA2B and ITGB3 as proteins but not as transcripts was due to contamination by platelets, we purified eosinophils by negative selection with antibody to ITGB3 in addition to the standard antibody “cocktail” described above. We then compared the proteomes of purified platelets, eosinophils purified by the standard method, and eosinophils purified with the extra depletion with anti-ITGB3 (1). ITGA2B and ITGB3, along with several platelet granule specific proteins, were outliers in a plot of ratios of standard eosinophils versus depleted eosinophils on one axis and platelets versus depleted eosinophils on the other. These data, which are compiled in our paper’s supporting XLSL file entitled “Summary of LFQ of eosinophil, platelets, and platelet-depleted eosinophils…” (1) can serve as a guide to assess platelet contamination in future proteomic studies of eosinophils.

## Phosphoproteome of Unstimulated and IL5-Stimulated Eosinophils

We used 10-plexed isobaric labeling to identify phosphorylation sites that change in eosinophils acutely activated with IL5. The advantage of isobaric labeling over LFQ is that if a phosphopeptide is identified, its relative abundance in relation to the same peptide in other samples can be estimated (34). Half of 20 × 10^6^ cells collected from each of five donors remained non-stimulated, and half were incubated for 5 min with IL5, 20 ng/mL. Incubations were stopped by plunging the tubes into liquid nitrogen. The 5-min stimulation induces maximal polarization and activation of MAPK1/3, STAT1, and STAT5 (17). When all 10 samples had been collected, cell pellets were thawed, cells were lysed, trypsin was added, and peptides from each sample were labeled separately with one of a 10-chemical set of tandem mass tags. Each of the 10 tags had the same mass, allowing the same peptides from multiple samples to be observed during the MS^1^ scan as a single *m*/*z* peak and isolated together for fragmentation. Each tag, however, had a unique distribution of heavy isotopes such that each tag yields a unique reporter ion upon fragmentation, and thus the relative amounts of the peptide in different samples can be determined based on relative intensities of the reporter ions in the MS^2^ scan. Peptides were enriched for phosphorylated peptides using IMAC before LC–MS/MS, and both unenriched and enriched peptides were analyzed. The Open Mass Spectrometry Search Algorithm (OMSSA) search algorithm was used along with our in-house software suite Coon OMSSA Proteomic Analysis Software Suite (COMPASS) (35, 36). Phosphorylation localization was performed with the Phospho RS 3.0 algorithm implemented into COMPASS. Statistical significance was determined using a two-tailed and equal variance *t*-test, *n* = 5.

Results from the isobaric labeling study were tabulated in Data Sheet in Supplementary Material entitled “Summary of proteins and phosphosites quantified in comparative analysis of unstimulated and acutely activated eosinophils” (1). The numbers of identified proteins (4,446) and phosphosites (1,819) were less than in the label-free analysis described above. The comparison of five individuals afforded the opportunity to assess individual-to-individual variation in protein abundances. The major differences were in HLA proteins. As would be expected given the short time of stimulation, only 16 proteins (0.3%) changed significantly (*p* < 0.001) between the resting and activated states. In contrast, 220 phosphorylation sites (12.1%) in 171 proteins changed significantly (*p* < 0.001) upon activation, 173 increasing and 47 decreasing (Table 1). Motif-X (37, 38) identified recurrent increased phosphorylation of Ser or Thr in motifs that are targets for MAPK or CAMIIK kinases.

**Table 1:** Summary of phosphorylation changes described in Sheet 2 of the Supplementary XLSX.

Process	Sites	Proteins	Up	Down	Notable examples
Chromatin	7	7	7	0	Known CDK2 site in BAP18
Replication	2	2	2	0	–
Transcription, templated	3	3	3	0	pS300 of LRRFIP1 up 22-fold
Transcription, general	11	10	9	2	pS43 of PBXIP up 12-fold
mRNA, splicing	17	14	10	7	4 decreased sites in SRRM2
mRNA, nuclear export	3	2	3	0	2 increased sites in ZC3H11A
mRNA, translation	9	8	5	4	pY233 of EIF4B up 13.6-fold
miRNA, processing	3	1	3	0	Single region of DDX17 helicase
Signaling, kinase	18	14	14	4	pS226 of MAP2K2 up 3.1-fold
Signaling, phosphatase	5	5	2	3	pY546 of PTPN11 up 20-fold
Signaling, scaffold	13	12	12	1	pS1134 of SOS1 up 6.4-fold
Signaling, small GTPase	21	19	17	4	S1834 of DOCK5 up 9.1-fold
Signaling, PI	7	6	5	2	pS1259 of PLCG2 up 9.1-fold
Signaling, ubiquitin	7	6	7	0	2 increased sites in HECTD1
Cytoskeleton, IF	11	5	10	1	6 sites in VIM including pY11
Cytoskeleton, microfilament	34	17	31	4	Multiple sites in EVL and RCSD1
Cytoskeleton, microtubule	7	7	4	3	pT154 of MAPRE1 up 3.1-fold
Vesicle-related	12	12	7	5	pT154 of PACS up 15-fold
Podosome-related	2	1	2	0	2 sites in BIN2
Membrane protein	5	5	2	3	pS405 of SELPLG tail up 5.9-fold
Metabolic	7	3	5	2	4 sites in NCF1
Unknown	16	12	14	2	5 sites in NHSL2

*The entries have been parsed for numbers of changed sites and proteins harboring the sites associated with each process and numbers of sites for which the phosphorylation increased or decreased. In addition, noteworthy examples of changes are given for all except one of the processes*.

*PI, phosphotidyl inositol; IF, intermediate filament*.

*In Sheet 2, the phosphorylated residues in FAM21B, LMNB2, PI4KA, and ARL6IP4 are renumbered compared with the entries for these proteins in original paper. The renumbering is in response to changes in the annotations of these proteins in UniProt*.

The top 18 most upregulated sites (8- to 25-fold increase) included phosphorylation of Tyr546 of tyrosine-protein phosphatase non-receptor type 11 (PTPN11, also known as SHP2) and Ser5 of plastin-2 (LCP1) both known to be critical early activation events in eosinophils (39, 40); Ser1259 of phospholipase C-γ2 (PLCG2); and Ser320 of p47^PHOX^ (NCF1), which controls activation of the respiratory burst oxidase (41). Sheet 2 lists all 220 sites with information about gene name, residue modified, fold change, *p*-value for the change, iBAQ intensity of the protein in the “global analysis” XLSX of our paper (1), name of protein, assignment of the protein to a single pathway or function, and implications of the phosphorylation. The last two determinations were made after inspection of information on the protein organized in UniProt and PhosphoSite.^4^ Significant changes were found in proteins that varied in abundance by as much as 22,000-fold. Only 13 of the significantly changed sites are unknown, i.e., not presently described in PhosphoSite. The proteins were assigned to 22 different processes (Table 1), and a notable example of a changed site is given for all except one category. The significance of the changed phosphorylation site varies from obvious to obscure. For instance, the increased phosphorylation of S226 of MAP2K2, the dual specificity kinase that activates MAPK1/3 (ERK2/1), involves one of the serines in the activation loop targeted for *O*-acetylation by *Yersinia* YopJ, a modification that inactivates MAP2K2 (42). In contrast, the four sites of decreased phosphorylation in SRRM2, a highly repetitive nuclear matrix protein involved in mRNA splicing, constitute a miniscule subset of the >280 sites listed in PhosphoSite as being phosphorylated in SRRM2. Changes in proteins associated with cytoskeleton were the most common but accounted for <25% of changed sites. Overall, the data are perhaps best interpreted as a snapshot at 5 min of a cell that is activated by IL5 to undergo simultaneous shape change, oxidative burst, new gene transcription, new mRNA processing and translation, and extensive shuttling of components among membrane compartments.

## Prospects for the Future

We have identified and quantified 7,086 proteins associated with non-activated peripheral blood eosinophils and demonstrated significant changes in 220 phosphosites in response to IL5 (1). For comparison, 10,225 proteins have been identified in HeLa cells (43), 7,952 in human embryonic stem cells (36), and ~4,200 in human platelets (44). Can the analyses of eosinophils be improved, what are the relative advantages of LFQ versus multiplexed isobaric labeling, and can such analyses lead to a better understanding of eosinophils in the context of eosinophil-associated diseases?

Our global data constitute a “version 1.0” of the eosinophil proteome that surely merits a “version 2.0.” More comprehensive coverage is important for finding peptides that define presently poorly characterized proteins and proteoforms such as ΔS and β variants of STAT3 described above and enabling one to exploit ongoing refinement of the human proteins-plus-proteoforms databases against which peptide sequence matches are made. Analyses of peptides generated by proteases other than trypsin such as chymotrypsin, LysN, LysC, AspN, and GluC can greatly increase % coverage of the sequences of identified proteins as well as increasing the number of identified proteins (12, 45). Such deep coverage requires preliminary fractionation of peptides and multiple LC–MS/MS analyses of the fractions. About 10^8^ purified eosinophils are needed, necessitating pooling of eosinophils from multiple donors inasmuch as only 2.5 × 10^7^ eosinophils will be purified from 200 mL of blood from a donor with a high normal eosinophil count of 250/μL if the yield is 50%. Our experience indicates that the analysis should be done on eosinophils purified by negative selection with antibody to ITGB3 in addition to antibodies to CD3, CD14, CD16, and glycophorin-A. We emphasize that no analysis will be ideal. The negative selections cannot remove other cell types completely, and the deeper one goes into the proteome the greater the chance of finding proteins from contaminating components of blood. In addition, workflows that avoid membrane-disrupting detergents, as ours does, may miss multipass membrane proteins with short loops and tails.

Prior proteomic studies have described alterations in amounts of eosinophil proteins in subjects with atopic dermatitis and eosinophilia (46), mildly elevated eosinophil counts associated with seasonal birch pollen allergy (47), and eosinophilia associated with *Fasciola hepatica* infection (48). In these studies, eosinophil proteins from affected individuals or healthy controls were separated by two-dimensional electrophoresis to produce high-resolution maps of protein-stained spots. The maps were compared by image analysis programs plus manual input, spots that stained differentially were identified, proteins in these spots were subjected to in-gel trypsinization, and tryptic peptides were identified by matrix-assisted laser desorption/ionization time-of-flight (MALDI-TOF) mass spectrometry. The numbers of proteins identified were considerably less than the number of spots subjected to trypsinization and MALDI-TOF because the same protein often was identified in multiple spots, presumably because of multiple proteoforms as described above or posttranslational modifications. One only has to examine the 2-dimensional maps upon which the quantification is based to realize the enormous amount of careful work that went into the studies. Nevertheless, the protein changes reported were not consistent, and the papers fail to identify a set of eosinophil proteins associated with increased eosinophil counts. The most complete study employing 2-D electrophoresis and MALDI-TOF identified 426 unique eosinophil proteins (20) as compared with the >7,000 and >4,400 that we identified in our LFQ and multiplexed isobaric labeling studies, respectively (1). With its vastly deeper coverage, ability to distinguish proteoforms and pinpoint post-translational modifications, standardized workflows, and intensity-based readouts that are amenable to facile statistical analyses, LC–MS/MS combined with LFQ or multiplexed isobaric labeling offers powerful and complementary approaches to the question of whether certain proteins in blood eosinophils are altered or predict therapeutic outcomes in patients with eosinophil-associated diseases.

The MaxLFQ algorithm, which is part of MaxQuant software suite, allows comparisons of protein abundance in different samples even though peptides from each sample are analyzed separately and the mix of quantifiable peptides from a given protein may vary from sample to sample (49). A recent study of individual variations in the 1,000 most abundant blood plasma proteins is an excellent example of the utility of LFQ (50). With 20 × 10^6^ eosinophils that can be purified routinely from individual subjects, it should be possible to perform LFQ of the ~5,000 eosinophil proteins that account for >99% of cellular molar abundance (1). One advantage of LFQ is the ability to analyze samples upon collection and, should data have clinical significance, communicate results within a clinically useful turnaround time. Multiplexed isobaric labeling would work well for comparisons of well-defined sets of subjects, such as those with mild versus severe asthma, for which samples could be archived over time and analyzed in batch. As above, the outstanding advantage of isobaric labeling is that the same peptide from all individuals will be detected and allow determination of the relative abundances of the peptide in the different individuals based on ion intensities in the reporter region (34). Being able to compare abundance of a given peptide in all individuals would be especially important in analyses of changes in specific phosphosites. The method suffers from contamination of the reporter region by reporter ions derived from co-isolated contaminating ions with resultant compression of ratios of reporter ion intensities (34). This problem, however, would lead to underestimation rather than overestimation of differences (51, 52). Once biomarkers are identified by either LFQ or isobaric labeling, it should be possible to devise a focused proteomic screen that employs multiple reactions monitoring for selected peptides with a set of these peptides labeled with heavy atoms serving as internal standards in an absolute quantification strategy (53, 54).

Planners of disease-oriented studies face the decision of whether to analyze eosinophils purified by the standard method, purified eosinophils also depleted of eosinophils to which platelets are adherent, or both types of purified eosinophils. Because platelets may modify eosinophil activity (31, 32), we favor not depleting eosinophils of eosinophil–platelet complexes, thereby not removing what may be the most interesting population of blood eosinophils. Abundances of proteins known to be specific for platelets can be used to estimate the contribution of platelets to observed proteomic differences.

A recent time course study using two-dimensional electrophoresis combined with MALDI-TOF to identify spots that stained differentially with a phosphoprotein-specific dye, ProQ Diamond, demonstrated that IL5-family cytokines increased phosphorylation of >20 eosinophil proteins in a pattern that was different from the effects of eotaxin or other agonists (55). Sites of phosphorylation were not determined. Multiplexed isobaric labeling should be a powerful method for pinpointing residues attacked by kinases or phosphatases upon eosinophil activation with different agonists and learning the effects of inhibitors and therapeutic agents on these phosphorylation events. We studied IL5 effects at only a single time point (1). Recently, 4,797 phosphosites were profiled temporally in an isobaric labeling study of platelets responding to ADP (56). The 1,819 phosphosites detected and quantified in our IL5 study are several-fold lower than in the platelet study. We believe that the latter number is achievable with eosinophils such that isobaric labeling studies can lead to a full “systems biology” understanding of the molecular events that underlie eosinophil activation in response to multiple agonists and how these events can be perturbed therapeutically.

## Author Contributions

All authors listed have made a substantial, direct, and intellectual contribution to the work and approved it for publication.

## Conflict of Interest Statement

The authors declare that the research was conducted in the absence of any commercial or financial relationships that could be construed as a potential conflict of interest.

## Footnotes

^1^http://www.uniprot.org/.

^2^http://mips.helmholtz-muenchen.de/corum/.

^3^https://www.proteomicsdb.org/.

^4^http://www.phosphosite.org.
